# Nonsyndromic Facial Asymmetry with Unilateral Condylar Aplasia

**DOI:** 10.1155/2013/631284

**Published:** 2013-08-18

**Authors:** Ranganadh Nallamothu, Rama Mohan Kodali, N. Koteswara Rao, Leela krishna Guttikonda, U. Vijayalakshmi

**Affiliations:** Department of Oral and Maxillofacial Surgery, Drs. Sudha & Nageswara Rao Siddhartha Institute of Dental Sciences, Chinoutpalli, Gannavaram, Krishna District, Andhra Pradesh 521 286, India

## Abstract

*Introduction.* The temporomandibular joint (TMJ) is the most complex elegantly designed joint in the human body. Abnormal development and growth of TMJ may lead to condyle aplasia present in several syndromes expressions, but it is extremely rare when not connected to any underlying pathological disorder or in conjunction with any syndrome. *Objective.* A rare case of aplasia of the mandibular condyle is presented, along with 3D computed tomography (3D CT) findings. *Conclusion.* Based on clinical and radiological findings we suggest the abnormal development of the TMJ as the origin. The 3D CT has provided high-quality images, which made diagnosis and a prompt treatment plan possible.

## 1. Introduction

The temporomandibular joint (TMJ) is the most complex elegantly designed joint in the human body [1]. It is a ginglymoarthrodial joint, a term that is derived from ginglymus, meaning a hinge joint, allowing motion only backward and forward in one plane, and arthrodia, meaning a joint which permits a gliding motion of the surfaces [2].

The right and left TMJ form a bicondylar articulation and ellipsoid variety of the synovial joints similar to knee articulation [3]. The most important functions of the TMJ are mastication and speech and are of great interest to dentists, orthodontists, clinicians, and radiologists. This interest stems from the standpoints of structure, function, adaptability, symptomatology, pathology, and imaging [4].

TMJ develops from separate temporal and condylar bronchial arches that grow towards each other at eighth week of fetal stage, with the ossification process starting at tenth week. The TMJ initial functions start at twentieth week during the fetal stage, when mouth opening movements appears it is before the development of the definitive joint. The development process will not be complete until twelfth year of life [5]. Varying degrees of condylar hypoplasia, from minimal to complete absence named as condylar aplasia, may occur due to abnormal development and growth of TMJ [5].

The most common causes of condyle alterations are inflammatory process in the area, rheumatoid arthritis, and radiotherapy [6]. The parathyroid hormone-related protein also affects the bone formation and chondrocyte differentiation and, consequently, the condyle formation [7, 8]. We are presenting a rare case of aplasia of the mandibular condyle, along with 3D computed tomography (3D CT) findings.

## 2. Case Report

**Figure 1:**
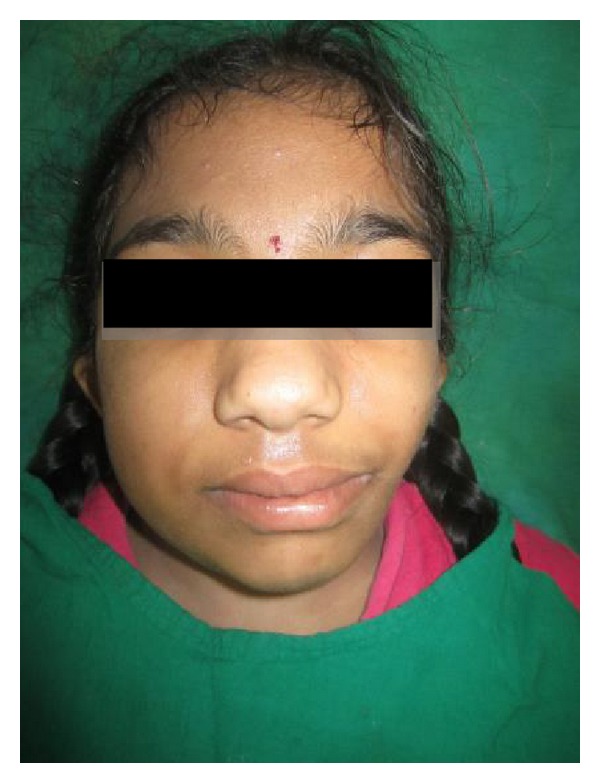
Frontal view.

**Figure 2:**
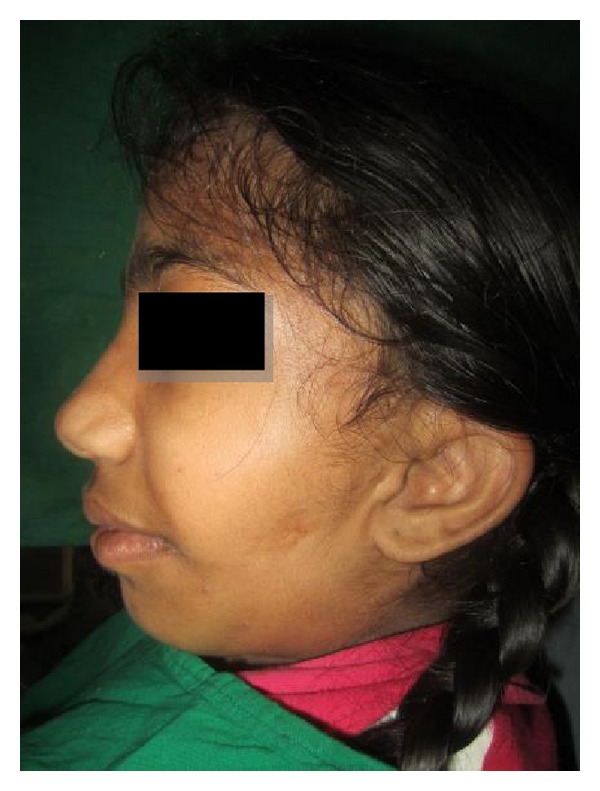
Lateral view.

**Figure 3:**
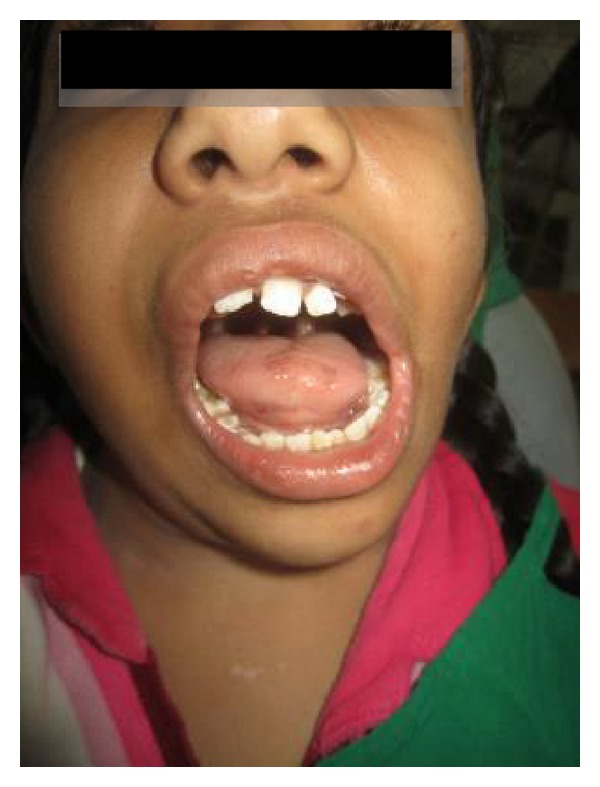
Deviation of the jaw to the left side.

A 10-year-old female presented with a chief complaint of asymmetry of left half of the face, which was first noticed during early childhood and had gradually progressed. At the anamnesis there was a history of forceps delivery. Her family history and personal history were noncontributory. No abnormalities were noted on general physical examination and her vital signs were within normal limits. Extraoral clinical examination revealed facial asymmetry (Figures 1 and 2) and affected side external ear (helix, antihelix, tragus, antitragus, concha scaphoid fossa, and lobule) was normal; a scar was noticed on the left side of the face and it was located well below the ala-tragal line and 1 to 2 cm away from the corner of the mouth, upon dwelling the history regarding scar it was revealed that patient gives history of fall while playing at school. Few months later the wound healed uneventfully and no developmental defects were noticed and there was a deviation of the mandible to the left side on opening (Figure 3).

**Figure 4:**
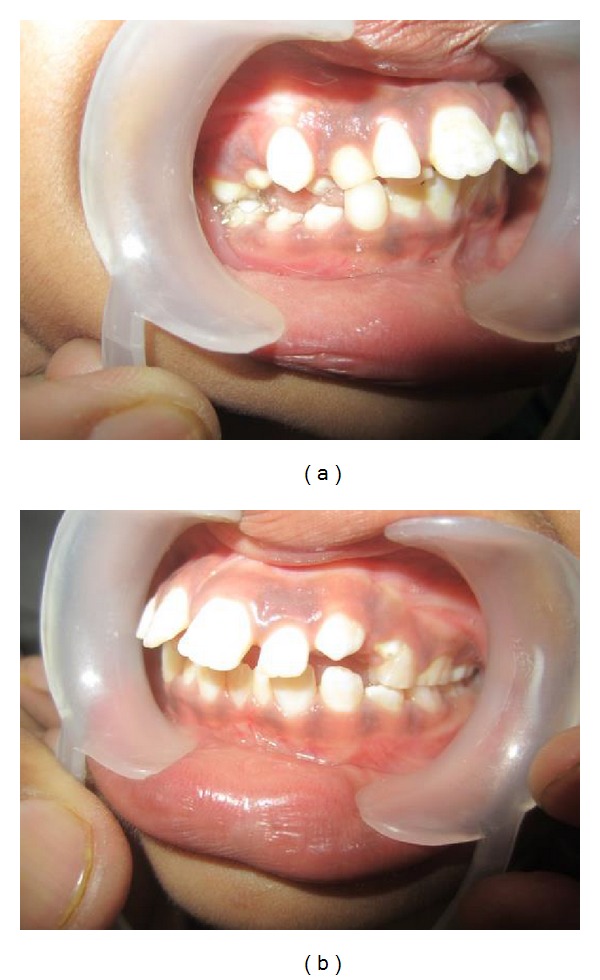
Intraoral right and left occlusal views showing mixed dentition.

**Figure 5:**
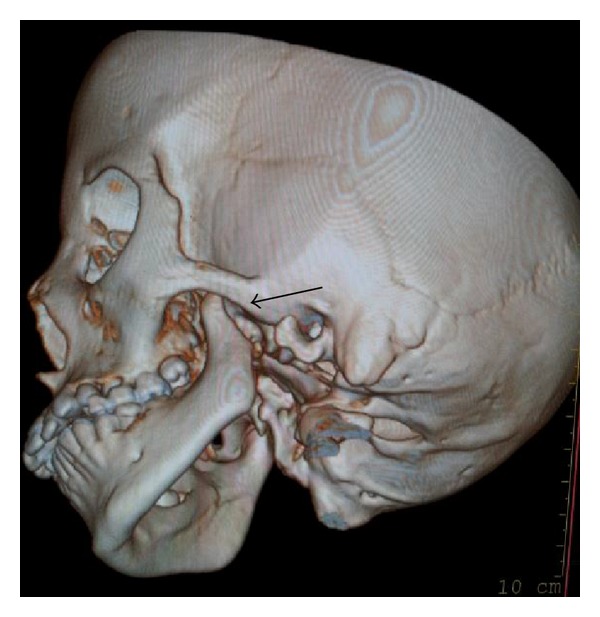
3D CT scan of affected side (left) with missing condyle and glenoid fossa.

**Figure 6:**
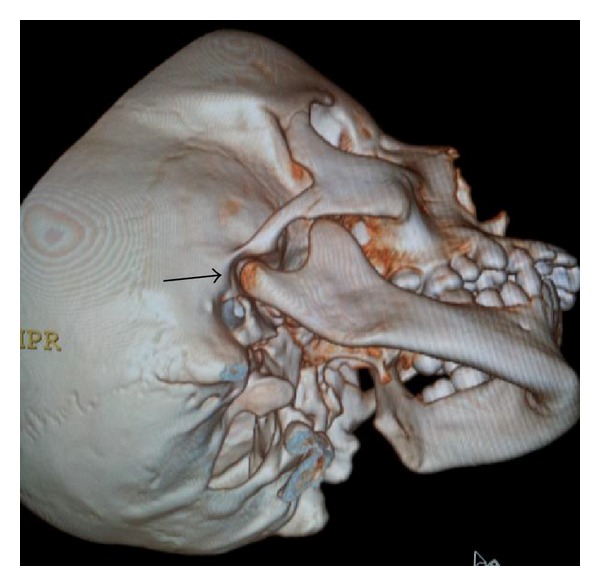
3D CT scan of normal side (right) with normal condyle and glenoid fossa.

On temporomandibular examination the left condyle is not palpable. Besides that no other important clinical findings were observed. Intraorally she had mixed dentition (Figures 4(a) and 4(b)) and hence the severity of malocclusion was not assessed. For detailed information, the patient was submitted to CT scan (3D). The images revealed complete condylar aplasia with missing condylar neck and glenoid fossa (Figures 5 and 6).

## 3. Discussion

Alterations, including condyle aplasia, are present in several syndromes expressions as observed in hemifacial microsomia, the Goldenhar syndrome, the Treacher Collins syndrome, the Proteus syndrome, the Morquio syndrome, and auriculocondylar syndrome. But the condyle aplasia is extremely rare when not connected to any syndrome [9, 10].

Facial asymmetry, deviation of the midline, and malocclusion are the consequences of the TMJ abnormality [9], as a syndrome expression (Figures 1 and 2). Our case shows the condyle aplasia without connection to any syndrome, and that highlights the rarity of the case.

The condyle malformation occurred due to abnormal development of the TMJ, probably at the fetal stage, before the tenth week. The trauma during delivery, as related, may not be the cause of the malformation in the present case; otherwise, there would not be a complete absence of the condyle, since its formation and ossification start at fetal stage. More often forceps delivery is correlated with TMJ ankylosis. With the arrival of 3D computed tomography (CT) and magnetic resonance imaging (MRI), diagnostic imaging of the TMJ has improved tremendously [11].

The 3D CT examination enables accurate surgical planning and provides quantitative information from skeletal and muscular parameters [12]. The present case needs a multidisciplinary approach including a team of maxillofacial surgeon, pedodontist, general surgeon, and orthodontist [13–15]. The treatment options for this situation are use of costochondral graft [16, 17], orthognathic surgery, and distraction osteogenesis [18, 19] based on the growth spurt concept.

Hence costochondral grafting procedure for the left temporomandibular joint was preferred before the growth spurt. Once the growth spurt ceases, either orthognathic surgery or the distraction osteogenesis will be preferred. Orthodontic treatment will be supported at all the stages of the treatment.

In summary, we report a rare case of unilateral condyle aplasia with no underlying pathological disorder or in conjunction with any syndrome. Based on clinical and radiological (3D CT) findings we suggest the abnormal development of the TMJ as the origin. Early recognition, accurate diagnosis, and multidisciplinary approach regarding the treatment will reduce the morbidity and provide better prognosis to these patients.
